# The complete chloroplast genome sequence of medicinal plant, *Artemisia argyi*

**DOI:** 10.1080/23802359.2016.1159926

**Published:** 2016-03-29

**Authors:** Sang-Ho Kang, Kyunghee Kim, Jeong-Hoon Lee, Byoung Ohg Ahn, So Youn Won, Seong-Han Sohn, Jung Sun Kim

**Affiliations:** a Genomics Division, National Academy of Agricultural Science, Rural Development Administration, Jeonju, Republic of Korea;; b Department of Plant Science, Plant Genomics and Breeding Institute, and Research Institute of Agriculture and Life Sciences, College of Agriculture and Life Sciences, Seoul National University, Seoul, Republic of Korea;; c Phyzen Genomics Institute, Seoul, Republic of Korea;; d Department of Herbal Crop Research, NIHHS, RDA, Eumseong, Republic of Korea;; e R&D Coordination Division, RDA, Jeonju, Republic of Korea

**Keywords:** Chloroplast, genome sequence, medicinal plant *Artemisia argyi*

## Abstract

*Artemisia argyi*, called wormwood, is widely distributed in northeastern Asia. The complete chloroplast genome sequence of *A*. *argyi* was generated by *de novo* assembly using whole genome next generation sequences. The complete chloroplast genome sequence of *A*. *argyi* is 151 192 bp in size. It is composed of a large single-copy (LSC), a small single-copy (SSC) and two inverted repeat (IR) regions of 82 930 bp, 18 344 bp and 24 959 bp, respectively. Overall GC contents of the genome were 37.46%. The *A*. *argyi* chloroplast genome has a total of 114 genes including 80 protein-coding genes, 30 tRNA genes and four rRNA genes. Phylogenetic analysis based on the chloroplast genome demonstrated that *A*. *argyi* is most closely related to *Artemisia montana*.

*Artemisia argyi* is a perennial herb as a member of the *Artemisia* belonged to the family Asteraceae (Bremer 1994) and widely distributed in Asia, such as China, Mongolia, Japan and Korea. *Artemisia* species are naturalized in dry and semi-arid habitats and mentioned as “wormwood”. *Artemisia argyi*, referred to as “Aeyup” in Korea, has been used for the treatment of colic pain, vomiting, and irregular uterine bleeding (Zhao et al. 1994). Despite its useful applications, there has been little report of molecular and genomic resources (Vallès & McArthur 2001; Shoemaker et al. 2005; Liu et al. 2013). In this article, the chloroplast genome sequence of medicinal plant, *A*. *argyi*, was completely characterized.

The plant samples of *A*. *argyi* were collected from Eumseong (Latitude: 36° 56′38.68″N, Longitude: 127° 45′17.60″E), Korea and identified by Dr. JH Lee, Department of Herbal Crop Research, NIHHS, RDA. A voucher specimen (MPS003336) is deposited at Korea Medicinal Resources Herbarium, Eumseong Korea. Whole genome sequencing was performed using Illumina genome analyzer (Hiseq1000, Illumina, San Diego, CA) platform at the in-house facility (Genomics Division, NAAS, RDA, Jeonju, Korea). *De novo* assembly was performed using CLC genome assembler (v. beta 4.6, CLC Inc., Aarhus, Denmark), as mentioned in Kim et al. (2015). We obtained three main contigs from *de novo* genome assembly. Main contigs cover the entire chloroplast genome of reported *A*. *frigida* chloroplast sequence (Liu et al. 2013). Three contigs could be joined as one single circular complete sequence by manual editing. Gene annotation was conducted using DOGMA (Wyman et al. 2004) and manual curation through comparison with published chloroplast genomes deposited on NCBI. Inverted repeats were identified using Tandem repeat finder (https://tandem.bu.edu/trf/trf.html). Simple sequence repeat (SSR) motifs were identified using NWISRL (http://ssr.nwisrl.ars.usda.gov/stop2.php). The complete chloroplast genome of *A*. *argyi* was submitted to GenBank under the accession no. KM386991.

The complete chloroplast genome of *A. argyi* is 151,192 bp in size, which is composed of a large single-copy (LSC), a small single-copy (SSC) and two inverted repeat (IR) regions of 82 930 bp, 18 344 bp and 24 959 bp, respectively. *A*. *argyi* chloroplast genome has a GC content of 37.46%. It contains 80 protein-coding genes, 30 tRNA genes and four rRNA genes. According to comparative analysis among three *Artemisia* species, sequence identities of whole chloroplast genomes were revealed like 99.8% between *A. argyi* and *A. montana,* otherwise, 99.3% between *A. argyi* and *A. frigida*. *A. argyi* was more closely related with *A. montana* than *A. frigida* according to analysis based on chloroplast genome. Simple sequence repeats (SSRs) of chloroplast genome sequence were found 92 in *A.argyi,* most of them were tri-nucleotide SSR motifs and commonly distributed throughout the genome.

The phylogeny of *A*. *argyi* to other species in the Asteraceae (family Compositae) was generated. A phylogenetic tree of *A*. *argyi* was constructed using entire chloroplast protein-coding sequences of *A*. *argyi* with 14 published species in family Asteraceae by a maximum likelihood (ML) analysis of MeGA 6.0 (MEGA Inc., Englewood, NJ) (Tamura et al. 2013). The phylogenetic tree indicated that *A*. *argyi* was grouped to the genus *Artemisia*, as expected and most closely related to *A*. *montana* in the genus *Artemisia* (Figure 1).

**Figure 1.**
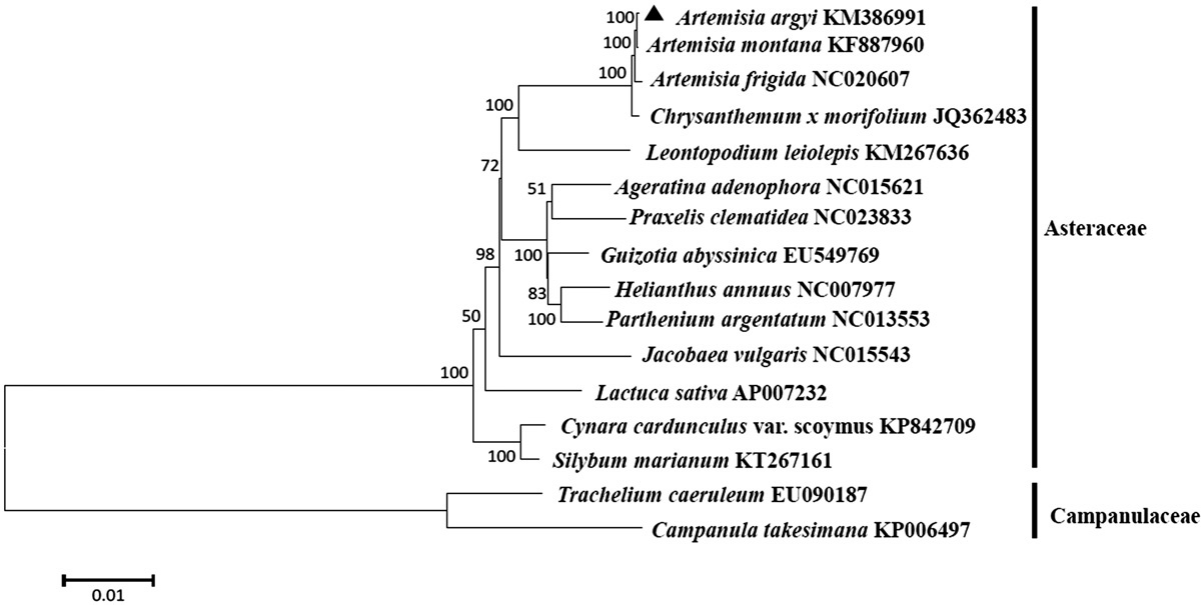
ML phylogenetic tree of *Artemisia argyi* with related 14 species of the family Asteraceae based on entire chloroplast protein-coding genes using Mega 6.0 with ML method. Numbers at the nodes are bootstrap values from 1000 replicates. *Trachelium caeruleum* (EU090187) and *Campanula takesimana* (KP006497) in Campanulaceae were set as the outgroup.

## Funding information

This work was carried out with the support of National Academy of Agricultural Science [Project no. PJ010889] and Cooperative Research Program for Agriculture Science and Technology Development [Project title: National Agricultural Genome Program, Project no. PJ010457], Rural Development Administration, Republic of Korea.
